# Editorial: Insights in cytokines and soluble mediators in immunity: 2022

**DOI:** 10.3389/fimmu.2023.1194553

**Published:** 2023-04-03

**Authors:** Serena Melgrati, Silvano Sozzani, Marcus Thelen

**Affiliations:** ^1^ Institute for Research in Biomedicine, Università della Svizzera italiana, Bellinzona, Switzerland; ^2^ Department of Molecular Medicine, Sapienza University of Rome, Laboratory Affiliated to Institute Pasteur-Italia, Rome, Italy; ^3^ IRCCS Neuromed, Pozzilli (IS), Italy

**Keywords:** cytokines, chemokines, inflammation, cell migration, cancer

Cytokines and chemokines are tightly regulated, promiscuous secreted proteins that regulate cell growth, differentiation, function and migration. These provide cues for immune cell trafficking and correct positioning in immune organs and tissues, and induce development of an immune response which is tuned to the immune insult. The main classes of cytokines include interleukins, interferons and members of the tumor necrosis factor (TNF). More than 40 interleukins have been presently identified, and may have distinct and overlapping functions (1). The TNF superfamily owes its name to the first member that was identified, TNF, that was initially found to induce necrosis in tumors (2, 3). This superfamily is composed of 19 proteins, some of which have pro-inflammatory and others anti-inflammatory properties. Chemokines consist of 47 small (8-10 kDa) proteins, which have the primary ability to induce directed cell migration (chemotaxis). Chemokines can bind to both canonical receptors, which couple to G-proteins and stimulate cell migration, and atypical receptors, which serve as scavengers to regulate chemokine availability (4, 5). Cytokines and chemokines have been implicated in numerous pathologies, including allergy, autoimmunity and tumor development and progression, and are often regarded as therapeutic targets.

This collection contains reviews on the evolution and role of one of the main interleukin superfamilies, IL-1; on the potential of TNFα inhibition in cancer therapy; and a systematic review and meta-analysis on the efficacy and safety of IL-13 inhibitors in atopic dermatitis. Moreover, two original research articles are present: a characterization of an isoform of CXCR3, which possesses an atypical function, and a study of chemokine gradient formation in 3D cell migration devices.

Boraschi discusses the evolution and role of the IL-1 superfamily and its receptors, analyzing their functions in homeostasis, innate and adaptive immunity and pathology. In particular, it is highlighted that IL-1/IL-1R link innate immune functions to the amplification and regulation of adaptive immunity. Some of the IL-1 family cytokines are only present in vertebrates or mammals, which suggests their requirement in promoting the efficacy of adaptive immune responses or mammal-specific needs such as pregnancy and embryonal implantation. It is proposed that the organ-specific activities and regulation has resulted in redundancy of the IL-1 family members. The non-immune, organ-specific functions of IL-1 and IL-1R, and the existence of further functions that do not depend on ligand-receptor binding are also described.

Ben-Baruch reviews the potential of TNFα in cancer therapy. The complexity of the TNFα-TNFR network is discussed, highlighting the controversial notion that these can lead to opposite effects in terms of cancer progression or tumor cell death depending on the context. This has therefore steered part of the scientific and medical communities to consider TNFα as a *target* by some, while it is regarded as a *therapy* by others. However, the possibility that these options might be interconnected is discussed: TNFα treatment might induce selection of cytotoxicity-resistant tumor cells, which leads to enhanced tumor progression. The author concludes by suggesting that highly specific, personalized approaches might pave the road ahead for better therapeutic strategies.

Zhang et al. reviewed the efficacy and safety of the interleukin-13 (IL-13) inhibitors lebrikizumab and tralokinumab in atopic dermatitis (AD). They found that both inhibitors reduced disease severity leading to improved quality of life, although urging for long-term studies to evaluate durability and to identify patients who would be more likely to respond to IL-13 inhibition. Overall, inhibiting IL-13 proves to be a valuable alternative for Th2-mediated AD to current therapies.

D’Uonnolo et al. analyze downstream signaling, subcellular localization and intracellular trafficking the two main isoforms of CXCR3: CXCR3-A and CXCR3-B. They demonstrate that the extended N-terminal domain of CXCR3-B confers it an “atypical” nature. Indeed, gradual truncation of this domain leads to increasing recovery of receptor surface expression and G protein coupling. They ultimately suggest that CXCR3-B could act as an atypical chemokine receptor for CXCR3 chemokines, which influences CXCR3-A activity by regulating the availability of such chemokines. Considering the role of IFNγ-induced CXCR3-triggering chemokines in the tumor microenvironment this is an interesting hypothesis on the mechanisms of the fate of immune responses (6).

Cell migration dynamics can now be easily studied using sophisticated 3D devices. Artinger et al. tackle the caveat that the electrochemical properties of chemokines are often neglected when utilizing fluorescent probes to assess gradient formation in 3D matrix migration chambers. They therefore generated site-specifically fluorescently labelled CCL19 and CCL21, which guide CCR7-mediated dendritic cell (DC) migration, and indeed reveal distinct gradient shapes for different chemokines, highlighting the limitations of using dextran as a surrogate marker. Furthermore, they identify that CCL21 in particular cannot freely diffuse within the 3D collagen matrix, contrarily to dextran, likely due to the polar C-terminal tail of CCL21, which interacts with extracellular matrix proteins. In contrast to dextran, the gradient of CCL21 possessed a steep shape, mimicking the *in vivo* situation where the CCL21 gradient sharply decreases away from the lymphatic vessel, which DCs migrate towards (7).

## Author contributions

SM wrote draft. MT edited draft. SS edited draft. All authors contributed to the article and approved the submitted version.
